# Rotary’s PolioPlus Program: Lessons Learned, Transition Planning, and Legacy

**DOI:** 10.1093/infdis/jiw556

**Published:** 2017-06-30

**Authors:** John L. Sever, Michael McGovern, Robert Scott, Carol Pandak, Amy Edwards, David Goodstone

**Affiliations:** 1 Rotary International, PolioPlus, Evanston, Illinois

**Keywords:** Rotary, volunteers, Polio eradication, lessons learned, transition planning & legacy.

## Abstract

Hundreds of thousands of Rotary volunteers have provided support for polio eradication activities and continue to this day by making financial contributions to the Rotary PolioPlus program, participating in national immunization days, assisting with surveillance, working on local, national, and international advocacy programs for polio eradication, assisting at immunization posts and clinics, and mobilizing their communities for immunization activities (including poliovirus and other vaccines) and other health benefits. Rotary has contributed more than $1.61 billion for the global eradication of polio and has committed to provide an additional $35 million each year until 2018 (all dollar amounts represent US dollars). Its unwavering commitment to eradicate polio has been vital to the success of the program. Rotary is providing additional support for routine immunization and healthcare. When polio is finally gone, we will have the knowledge from the lessons learned with PolioPlus, such as the value of direct involvement by local Rotarians, the program for emergency funding, innovative tactics, and additional approaches for tackling other global issues, even those beyond public health. Rotary has already transitioned its grants program to include 6 areas of focus: disease prevention and treatment, water and sanitation, maternal and child health, basic education and literacy, economic and community development, and peace and conflict prevention/resolution. Funding for these grants in 2015–2016 was $71 million. The legacy of the polio program will be the complete eradication of poliovirus and the elimination of polio for all time.

Rotary is a global, nonprofit organization of 1.2 million business and professional leaders spread across over 35000 clubs in some 200 countries and geographic regions. Rotary members are committed to providing service to their communities and the world. Their primary motto is “Service Above Self.” 

## ROTARY’S ADOPTION OF POLIO ERADICATION AS A GOAL

In 1978, Rotary expanded its programs from local service activities and international scholarships to include national and international projects in “Health, Hunger, and Humanity” (3-H). Clem Renouf, president of Rotary International from 1978 to 1979, had read about the eradication of smallpox, which was recent proof that a disease could be wiped out. He wanted a similarly ambitious project to initiate Rotary’s new 3-H projects [1, pp 47–50]. He asked a Rotary member who was a medical authority on infectious diseases if Rotary might take on another disease with similar results. The Rotarian advised him that if a single vaccine were selected for the 3-H program he would recommend polio. Infection by poliovirus was a major public health problem that caused hundreds of thousands of children to be permanently paralyzed or die each year. An oral vaccine was available that could prevent polio and was easy to give. Renouf accepted the advice and proposed polio eradication to Rotary’s Board of Directors. Later in 1979 the Board responded and adopted “the eradication of poliomyelitis and the alleviation of its consequences” as a primary 3-H goal of Rotary [1, p 47].

The first 3-H Grant was for $760000 to immunize 6 million Philippine children against polio in 1979. At that time the Philippines had the highest incidence of polio in the western Pacific [1, pp 47–50]. It was a good location for Rotary to attempt a national polio immunization project. The Philippines had a strong Rotary club presence to mobilize communities, good coordination between the government, other important institutions, and nongovernmental organizations (NGOs), and a past secretary of health belonged to the Rotary Club of Manila [1, p 47].

The project began in September 1979, and the success of this 3-H immunization project expanded into a polio eradication program on an unprecedented scale. In February 1982, Rotary adopted the goal of immunizing all of the world’s children against polio by the time of the 100th anniversary of Rotary International, in 2005 [2]. Additional Rotary grants were approved for polio vaccine for use in existing vaccination operations in Haiti, Bolivia, and other countries [1, pp 63–5].

In 1984–1985, Rotary assembled a special committee to work with expert advisors, including Albert Sabin (now deceased), MD, developer of the oral polio vaccine and Alan R. Hinman, MD, MPH of the US Centers for Disease Control and Prevention (CDC) to develop an expanded Rotary program for the eradication of polio [1, p 70]. That program included the use of national immunization days for mass polio vaccination and greatly increased funding from Rotary, with an initial fundraising goal of $120 million for polio vaccine. Within 3 years, Rotarians had raised more than double their fundraising goal, donating $247 million [1, p 96]. The program was named PolioPlus, and the “Plus” recognized the importance of other childhood vaccinations as part of primary healthcare and reflected the strategy to provide other health initiatives along with polio.

### Lessons Learned

It was essential that Rotary selected polio, a global health problem of recognized international importance for which a vaccine was available. Timelines were set for completion of the program but these had to be revised as various events delayed progress. Rotary’s unwavering commitment to complete the eradication of polio has been vital to the success of the program. In the words of Dr Margaret Chan, Director-General of the World Health Organization (WHO): “Rotary International is the top private sector contributor and volunteer arm of the eradication initiative. The 1.2 million Rotarians envisioned a polio-free world, and then challenged governments and health agencies to pursue this vision” [3].

## SPEARHEADING PARTNERS, EXPERTS, COORDINATION, AND THE GLOBAL POLIO ERADICATION INITIATIVE

Rotary knew that eradicating a disease like polio, which in the late 1980s was endemic in 125 countries, required cooperation with other major organizations [4]. Therefore, in 1988, after adopting a World Health Assembly Resolution on the “global eradication of poliomyelitis,” WHO, the United Nations Children’s Fund (UNICEF), and the US CDC joined Rotary as the spearheading partners of a new public-private partnership called the Global Polio Eradication Initiative (GPEI) [5, 6]. The Bill & Melinda Gates Foundation also became a core partner with the GPEI and a major supporter. Other key players include most of the world’s national governments, corporate partners, NGOs, and individual donors.

Rotary enlisted the help of technical experts including, for example, Albert Sabin and Ciro de Quadros (now deceased), MD, MPH (head of the immunization program for the Pan American Health Organization) to develop the most effective strategies for the initiative. Public health authorities from the WHO, UNICEF, and the US CDC continue to be regular advisors to Rotary. The development of interagency coordinating committees—which include representatives of the relevant ministries of health, donor agencies for each country, core partners, and other stakeholders—defined the roles of the partners.

Rotary has also played important leadership roles in eradication campaigns. For example, Rotary chaired the interagency coordination committee that included the WHO, UNICEF, the US CDC, the US United States Agency for International Development (USAID), and other local partner organizations in Operation MECACAR (Mediterranean, Caucasus and Central Asian Republics), a polio eradication campaign from 1995 to 2000 [7, pp 87–90]. Rotary is currently the co-chair of the interagency coordination committee for the African region.

### Lessons Learned

In creating the PolioPlus program, and later serving as a spearheading partner of the GPEI, Rotary proved the value of a civil society organization in effective coalition building for great impact. The participation of expert advisors is essential to meet the changing needs of the program.

Rotary’s work with core partners in eradicating polio has demonstrated the importance of well-coordinated partnerships for global health. During Operation MECACAR, Rotary’s work on advocacy, financial support, and assisting the European laboratory network was complemented by other core partners. WHO provided technical guidance, and UNICEF aided the effort through “social mobilization activities” and its “ability to procure vaccine of high quality at low cost and deliver it in a timely fashion” [7, p 89].

## THE ROLE OF ROTARY MEMBERS

From the beginning of the polio eradication program (and even long before the formation of the GPEI), Rotary members have been actively participating in polio eradication efforts. They use their business acumen and passion for volunteerism to raise funds, build awareness, and encourage national governments and others to donate to and otherwise support the polio eradication effort.

Rotary members have provided $1.61 billion, and they have committed to raise and spend $35 million annually until 2018. Their current contributions are matched 2 to 1 by the Gates Foundation, so this will secure $105 million yearly through 2018. True to the goals of PolioPlus, Rotary members have also taken part in immunization campaigns and broader health interventions and assisted with social mobilization and surveillance efforts.

In Nigeria, Rotarians have sponsored and assisted at health camps set up to provide multiple medical services, including immunization against polio and other communicable diseases, such as measles, the treatment of parasitic infections, and the provision of bed nets to protect against malarial mosquitoes [8]. In India, Rotary partnered with a global healthcare company to provide “Mega Wellness Camps,” where a large number of physicians “provided free consultation and education to all walk-in patients on a wide range of health issues” [9].

Rotary members are the grassroots agents of change, helping to bridge cultures to reach every community with the vaccine. By working with others to engage community and religious leaders, they enable health professionals to do their work. In addition, they have participated in a large number of national immunization days. With this hands-on experience, they are able to share their accounts of immunizing children to help educate their communities and further raise awareness and funds.

They have also participated in humanitarian diplomacy to overcome hurdles in reaching every community with the polio vaccine. For example, during the Sri Lankan civil war, the planned national immunization days were affected by the fighting. The government planned to target only areas not affected by the war, which meant that only two-thirds of the country’s children would be vaccinated. Rotary, in collaboration with UNICEF, was able to engage the rebel army leader in negotiations. They managed to secure a ceasefire agreement between the government and the rebels, and the subsequent vaccinations reached approximately 95% of the country [10]. This tactic of humanitarian diplomacy, known as “days of tranquility,” is used in other countries, so that polio eradication programs are not disrupted by conflict [11].

Rotary has engaged in specialized and general social mobilization. For example, in Operation MECACAR, Rotary also helped to implement “a successful approach to the Roma minority communities of Bulgaria and Romania” and convinced minority ethnic groups to participate in national immunization days—despite these groups’ deep-seated distrust of government programs” [12]. Rotary’s social mobilization strategy “brought many previously reluctant parents forward to vaccinate their children for the first time,” and brought the vaccine to communities across the Mediterranean, Caucasus, and Central Asian Republics [7, pp 87–90]. The diplomatic efforts of Rotary members at the highest echelons have also borne fruit; “Rotary had the legitimacy to convince the Kosovo Albanian leadership to accept vaccination of their children by the Serb health authorities”[7].

The influence of Rotary members extends not only to social mobilization but also to delivering the vaccine across international borders and in remote regions. Because of its nongovernmental status, Rotary has played a key role in cross-border immunization efforts—for example in “Operación Limpieza” (Operation Mop-up) in Ecuador, Peru and 7 other Latin American countries in the late 1980s [13]—and in the delivery of vaccine today to communities in the border area between Pakistan and Afghanistan [14].

### Lessons Learned

Rotary’s global reach, the reputation of Rotary members as credible, impartial interlocutors, and their tenacity in the pursuit of polio eradication all have helped drive the success of PolioPlus. Rotary’s deep roots as a civil society organization allowed it to reach more communities, cross cultural bridges, and bring the vaccine to as many children as possible despite obstacles of politics and war. The direct involvement of local Rotary members who speak the language has helped facilitate countless polio immunization campaigns in polio-affected countries. United Nations Secretary-General Ban Ki-Moon recently praised the “monumental contributions to eradicating polio” of Rotary members [15].

## GRANTS WITH REVIEW AND PERFORMANCE MONITORING

Rotary established a grants program to review, approve, and monitor funding proposals from WHO, UNICEF, and Rotary programs. Grant proposals are first reviewed by national and regional PolioPlus committees and then by a grants subcommittee of the Rotary International PolioPlus Committee (IPPC), before presentation to the full committee. The IPPC meets 3 times each year with advisers from the WHO, UNICEF, and the US CDC to review the grants. Grants that are recommended for funding are forwarded to the trustees of the Rotary Foundation for final consideration and approval. Monitoring of performance is conducted by the IPPC and includes reviews of progress reports for grants that have been funded. Financial reviews of expenditures by grant recipients are conducted periodically, and special investigations are instituted as needed.

**Figure 1.**
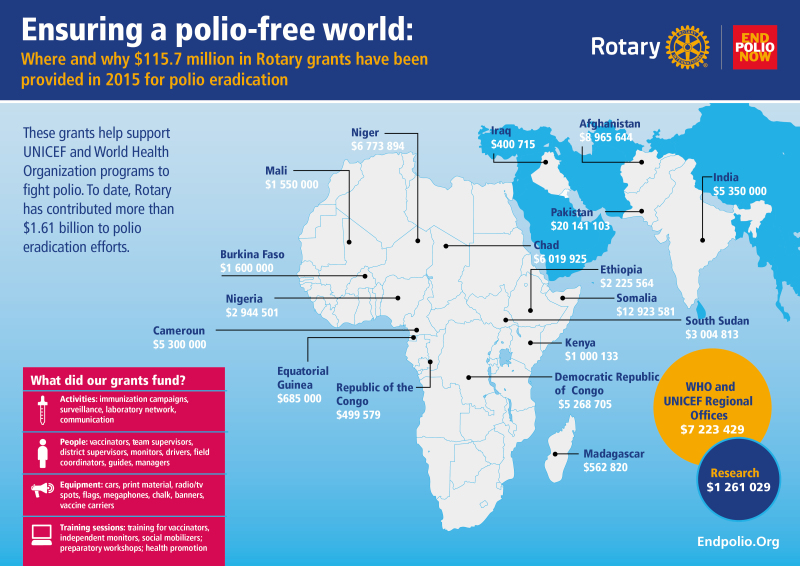
Distribution of Rotary grants in 2015. This is reprinted with permission from Rotary International. Abbreviations: UNICEF, United Nations Children’s Fund; WHO, World Health Organization.

In 2015 Rotary supported polio eradication activities in 18 countries with grants of $115.7 million (Figure 1) ($35 million from Rotary, a matching grant of $70 million from the Gates Foundation, and a supplemental Gates grant from an earlier fundraising effort). Activities that were funded included, for example, advisors, training, vaccines, transportation, surveillance including the laboratory network, communication, social mobilization, high-quality immunization through national and supplemental programs, and immunization posts (Figure 1).

Rotary also awards rapid-response grants of $500000, which can be requested by the chair of the IPPC and authorized by the chair of the trustees for immediate funding of special polio emergencies. For example, in 2013, a Rotary rapid-response grant was provided to the WHO for Somalia to help respond to a polio outbreak in the Horn of Africa. It covered “operational costs, including human resources, training, and transportation of health workers, to immunize children under 10 in all accessible areas” of the country [16]. A total of 10 rapid-response grants have been awarded in the past 10 years. This type of unique flexibility has helped the program greatly in Somalia, Myanmar, and Sudan, and recently in the Middle East, to help prevent a polio outbreak in Iraq and Syria in 2013 from spreading further [17].

### Lessons Learned

The GPEI is a project of great financial complexity, which requires significant administrative support to manage at the international and local levels. Taking steps to ensure financial accountability is an important lesson of the PolioPlus program. Rotary instituted a rigorous grants review process, with the ability to conduct special investigations of expenditures to ensure the accountability, transparency, and integrity of the grants process.

Rotary’s emergency funding mechanism of up to $500000 allows the chair of the IPPC, with the concurrence of the chair of the trustees, to provide special grants for emergency situations that would not otherwise be possible. These grants also act as a catalyst, encouraging governments and other donors to fund emergency responses.

## PROGRAM IMPLEMENTATION AND INNOVATIVE STRATEGIES

In the field, Rotary members have been innovative in supporting polio surveillance activities and ensuring high quality immunization campaigns. For example, Rotary’s current PolioPlus strategy in Pakistan—the country where the majority of polio cases are now being reported—includes 5 core elements: advocacy, health camps, permanent transit posts, permanent immunization centers, and Rotary polio resource centers. They are the building blocks of Rotary’s concerted effort to interrupt transmission of the virus.

Permanent immunization centers along the shared border between Pakistan and Afghanistan have helped reach children in migratory populations. They also provide a safe place for both the vaccinators and the children, in the context of ongoing conflict. Similarly, strategically placed permanent transit posts at entry points to international borders, provinces, and big cities across Pakistan have reached mobile populations with the vaccine.

Also in Pakistan, Rotary has trained female health workers in the use of cell phone data reporting, which allows real-time recording of immunization coverage and public health surveys of populations, helping to focus polio immunization drives. The national chair notes how “Lady Health Workers are trusted to enter households and have the interactions with mothers and children necessary to deliver the polio vaccine. They also provide education and services for antenatal care, maternal health, and routine immunization for other diseases, such as Hepatitis B, Tetanus and measles” [18].

In Nigeria and India, Rotary has put on health camps to create demand for polio vaccinations, and worked with national groups, such as the Federation of Muslim Women’s Associations. By working with traditional and religious institutions, these activities are designed to promote local ownership and accountability at all program levels, deepen community involvement, and increase uptake of the oral and inactivated polio vaccines [19].

Other countries have used different tactics to draw in local populations and enhance PolioPlus activities. Rotary members in South America distributed posters and coffee mugs with dedicated phone numbers to report cases of acute flaccid paralysis as part of surveillance strategies. In the Democratic Republic of Congo, billboards are erected with a schedule for routine immunizations.

### Lessons Learned

Innovative tactics, like the creation of permanent transit posts in Pakistan, have been instrumental in reaching mobile populations, and immunizing frequently missed children. The community-based volunteer vaccinator training program has unleashed the power of volunteerism to engage local populations and maximize the workforce fighting against polio. Again, one of the strengths of this initiative is the clear definition of roles for each partner. Recent PolioPlus grants supported WHO operations and staff in Cameroon and Ethiopia, as well as support for UNICEF’s social mobilization activities in Chad and Nigeria. The activities funded include the training and salary of hundreds of thousands of vaccinators, and >100000 picture books in local languages describing the effects of the poliovirus and benefits of polio immunization [20].

## INTERNATIONAL ADVOCACY

Rotary has leveraged the power of constituents in both polio-affected and donor countries. The only partner in the GPEI with 1.2 million members who are citizens in some 200 countries and geographic regions, Rotary is well positioned to urge officials from the local to national level to focus on polio eradication.

Very active advocacy programs have provided significant financial support and resources from donor governments, industry, and individuals. Advocacy has also been important for enlisting the support of governments where polio eradication programs are in progress.

At the national level, PolioPlus summits in India and Nigeria helped secure ongoing political commitment from heads of state. In Pakistan, Rotary’s national PolioPlus Committee chairman met with the governor of Balochistan in Quetta to request strengthened military support at border areas to protect health workers and ensure wider vaccination coverage of children <5 years old traveling between Afghanistan and Pakistan [21].

More than $11 billion has been invested in the GPEI by donor and polio-affected governments, private sector partners, NGOs, and developments banks because of strong advocacy by Rotary and other groups. Such advocacy has also achieved important declarations to bolster and extend support for eradication, from the United States, the European Union, the African Union, the Organization of the Islamic Conference, the Commonwealth group of countries, and many others [22–25].

Advocacy at local and national levels has also helped overcome resistance to the polio vaccine. In Pakistan, India, and Nigeria, for example, Rotary and its partners appealed to specialist bodies of Islamic scholars (Ulemas) to support polio vaccination, and provided training on polio and its impact. National Islamic leaders have issued fatwas in support of the vaccine, promoting its safety. Rotary members have then printed these fatwas and distributed them to vaccinators for circulation in communities.

### Lessons Learned

Rotary’s credibility as a nonpartisan NGO has aided its advocacy efforts. In the words of one Rotary member, “[We learned] how to leverage Rotary’s comparative advantage as a civil society voice that, in some cases, is able to make pointed statements that international organizations would not be able to make… We are the only partner that is constituent-based, made up of citizens who are living in the countries whose support is being sought… This has made a huge difference in ensuring polio remains on the agenda*”* (Anonymous, personal communication). Rotary can hold governments accountable to follow up on funding commitments, and encourage them to provide the political support necessary for polio immunization programs.

## COMMUNICATION FOR GENERAL AWARENESS AND SUPPORT

Special communication programs have been conducted and are ongoing to maintain awareness of polio, particularly in countries that no longer suffer from the virus, and to combat any potential mission fatigue. Rotary’s “This Close” public awareness campaign enlisted celebrities and other major public figures to promote Rotary’s End Polio Now campaign. The Public Service Announcements and print advertisements featured the tagline “We’re this close to ending polio,” and each participant is shown with the signature “this close” gesture with their thumb and forefinger in close proximity. The campaign highlights the progress made in reducing the global number of cases of polio by 99.9% and encourages the public to support efforts to stamp out the final 0.1% of cases [26]. The campaign continues to be a great success, and Rotary surpassed its initial target of $200 million, raising $228.7 million in 2012, which led to a further funding partnership by which the Gates Foundation matches 2 to 1 any contributions made by Rotary members, up to $35 million per year through 2018.

Another significant communications strategy for Rotary’s PolioPlus campaign has been the illumination of iconic structures across the world, from the United Kingdom’s Houses of Parliament to the pyramids of Egypt with the End Polio Now message on the organization’s 108th anniversary in 2013 [27]. Other innovative polio communications initiatives are now Guinness world records. For example, Rotary created the “world’s biggest commercial” when >100000 persons from 171 countries posted selfies in support of End Polio Now (2014), and the world’s largest human national flag, composed of 50000 persons, in Chennai, India (also in 2014) [26, 28].

### Lessons Learned

Targeted communications campaigns, particularly in countries that no longer have polio cases, have been vital to maintain support for PolioPlus. An ambitious goal required Rotary to use creative approaches to galvanize Rotary members and others into action around a common cause. Quantifying the economic and humanitarian impact of polio eradication makes a compelling case, but even so, new initiatives were required to keep polio eradication relevant in countries that no longer had any new cases to avert donor fatigue. For example, the incentive of a 2-for-1 match by the Gates Foundation re-energized the contributions of Rotary members, even after many decades of dedication to the campaign.

Rotary also saw the value in recognizing donors, whether individuals, institutions, or governments as a way to maintain engagement in PolioPlus. When PolioPlus began, a recognition program was also started as part of the fundraising campaign, supporting the concept that PolioPlus was an extraordinary program and those who contributed should be recognized accordingly. Different “recognition packages” were linked directly to 6 levels of contributions, ranging from $1000–4999, to $250000 plus [29].

The recognition program evolved to highlight and encourage new champions of the cause. The Polio Eradication Champion Award recognizes governmental leaders for their support of Rotary’s flagship project. Past recipients of the Rotary award include Bill Clinton, president of the United States; Angela Merkel, Chancellor of Germany; Prime Minister David Cameron of the United Kingdom; Prime Minister Tony Abbott of Australia; United Nations Secretary-General Ban Ki-moon; and 48 members of the 114th US Congress [30].

## TRANSITION PLANNING

In 2013 Rotary transitioned its 3-H grants to a new global grants program for Rotary clubs, which defines 6 areas of focus for grant awards: disease prevention and treatment, water and sanitation, maternal and child health, basic education and literacy, economic and community development and peace and conflict prevention/resolution. In 2015–2016, this program approved 1082 grants and awarded $71 million of funding, with an emphasis on sustainable investments driven by community-based needs assessments, local advocacy, and community engagement, all lessons learned from the ongoing polio eradication program.

For example, every year (since 2012) a special Rotary member group focused on family health and AIDS prevention holds family health days in Africa [31, 32]. Rotary members from 365 clubs fan out across Uganda, Nigeria, and South Africa to help medical professionals and government workers provide free health services to 250000 persons. The event includes polio and measles immunizations, dental and eye clinics, and family counseling and screening for HIV, diabetes, hypertension, and breast and cervical cancer.

Rotary is developing a transition plan to ensure that all the resources of polio eradication—both the physical infrastructure and the human knowledge acquired—can be transferred to other health priorities. Rotary’s unwavering commitment to eradicate polio has been vital to the success of the program. Rotary’s primary goal remains polio eradication, and this has been increasingly integrated with actions to improve health systems, support routine immunization for other diseases, and boost health worker capacity. For example, as part of the disease prevention and treatment area of focus, Rotary aims to improve and expand access to low-cost and free healthcare in underdeveloped areas.

## LEGACY

The most important legacy of this program will be the eradication of polio. This will provide eternal benefit to the people of the world by eliminating this serious, damaging, and deadly infectious disease for all time.

Through Rotary’s polio eradication efforts, the organization has learned how to raise funds with coordinated campaigns, and raise awareness with innovative communications methods and celebrity engagement. In addition, Rotary members learned how to work with other organizations to implement large-scale projects that required advocacy, security planning, and coordination in the field. Finally, the launch of PolioPlus as Rotary’s flagship project has a legacy of uniting Rotary members around the world behind a common goal. Ending polio will be a major stepping stone for the United Nations’ sustainable development goals, particularly goal 3, to “ensure healthy lives and promote well-being for all at all ages” [33]. When polio is finally gone, we will have the blueprint for tackling other global issues, even those beyond public health.

## Notes

***Acknowledgments.*** We thank Saujanya Gumidyala and Avery Mamon of Rotary International for their kind assistance in preparing Figure 1.

***Supplement sponsorship.*** This work is part of a supplement coordinated by the Task Force for Global Health with funding provided by The Bill and Melinda Gates Foundation and the Centers for Disease Control and Prevention.

***Potential conflicts of interest.*** All authors: No reported conflicts. All authors have submitted the ICMJE Form for Potential Conflicts of Interest. Conflicts that the editors consider relevant to the content of the manuscript have been disclosed.
